# Preclinical testing of a novel personalized cancer vaccine for all solid tumors and all patients

**DOI:** 10.1186/2051-1426-3-S2-P208

**Published:** 2015-11-04

**Authors:** Doreen Jackson, Julia M Greene, Diane Hale, Erika Schneble, Thomas Wagner, Xianzhong Yu, George E Peoples

**Affiliations:** 1 San Antonio Military Medical Center, Converse, TX, USA; 2 San Antonio Military Medical Center, San Antonio, TX, USA; 3 USAF, Colorado Springs, CO, USA; 4 Perseus, Greenville, SC, USA; 5 Perseus/Clemson University, Greenville, SC, USA; 6 Cancer Vaccine Development Program, San Antonio, TX, USA

## Background

A variety of autologous tumor/dendritic-cell (DC) vaccines have been pursued. Our prior autologous tumor/DC fusion (dendritoma) vaccine demonstrated clinical benefit in metastatic melanoma; however, dendritoma production is difficult and not scalable for commercialization. We developed an alternative, novel approach to efficiently deliver the autologous tumor antigenic repertoire to the cytoplasm of phagocytic-DC using yeast cell wall particles (YCWP). We describe the *in vitro* and animal data supporting clinical development of our tumor lysate, particle-loaded, DC(TLPLDC) vaccine.

## Methods

YCWP are prepared, leaving beta glycan hollow spheres with large pores capable of loading. TL is produced from multiple freeze/thaw cycles and loaded in YCWP. Monocyte-derived DC are isolated and matured. Using the ovalbumin/B3Z cell system, spectrophotometric analysis confirmed antigen presentation via YCWP vs exogenous loading. *In vivo* effectiveness of TLPLDC compared to dendritomas and controls was assessed in a murine melanoma pulmonary metastasis model (MMPMM). DC were prepared from mouse bone marrow, and YCWP loaded with TL from the B16F0 murine melanoma cells. Treatment group mice were inoculated intradermally with 1×10^6^ TLPLDC one week prior to tumor challenge and repeated for three weekly doses. Controls were inoculated similarly with saline, irradiated tumor cells, empty DC, fusion mixtures or dendritomas. All mice were injected intravenously with 5×10^5^ B16 tumor cells. Upon death of the first animal, all were sacrificed, lungs harvested, and pulmonary metastases totaled. In another comparison, untreated control animals were compared to TLPLDC-treated with and without CpG adjuvant for survival. Statistical analyses were performed using t-test or ANOVA as indicated.

## Results

Based on spectrophotometric analysis, the ovalbumin loaded YCWP-fed DC produced a 100-fold increase in epitope-specific CD8+ T cell responses compared to traditional exogenously loaded DC. In the MMPMM, TLPLDC inoculations resulted in zero metastases compared to saline (198+1.5 mets), irradiated tumor cells (>200), empty DC (>200), fusion mixture (112+25), and dendritoma (3.13+1.5) controls(n=8, each; all p < 0.01). Median survival of untreated controls was 21 days, TLPLDC inoculated was 29 days and TLPLDC+CpG inoculated was 41 days (n=10, each).

## Conclusions

YCWP are more effective than exogenous antigen loading of DC and produce a robust CD8+ T cell response. In the virulent B16 murine melanoma model, TLPLDC, and particularly TLPLDC+CpG, produced long-term survival and out-performed our previous dendritoma vaccine which demonstrated clinical benefit in Phase I/IIa trials in metastatic melanoma patients. This data bridges our previous tumor/DC fusion technology to the novel TLPLDC and supports clinical development of the TLPLDC vaccine.

